# Retrospective analysis of the incidence and outcome of late acute and chronic graft-versus-host disease—an analysis from transplant centers across Europe

**DOI:** 10.3389/frtra.2024.1332181

**Published:** 2024-03-18

**Authors:** Ronja Langer, Antonela Lelas, Michael Rittenschober, Agnieszka Piekarska, Alicja Sadowska-Klasa, Ivan Sabol, Lana Desnica, Hildegard Greinix, Anne Dickinson, Marit Inngjerdingen, Anita Lawitschka, Radovan Vrhovac, Drazen Pulanic, Sibel Güneş, Stefan Klein, Jan Moritz Middeke, Matthias Grube, Matthias Edinger, Wolfgang Herr, Daniel Wolff

**Affiliations:** ^1^ Department of Internal Medicine III, Hematology and Oncology, University Hospital Regensburg, Regensburg, Germany; ^2^ Division of Hematology, Department of Internal Medicine, University Hospital Centre Zagreb, Zagreb, Croatia; ^3^ St. Anna Children’s Hospital, Medical University of Vienna, Vienna, Austria; ^4^ Department and Clinic of Hematology and Transplantology, Medical University of Gdańsk, Gdańsk, Poland; ^5^ Division of Molecular Medicine, Rudjer Boskovic Institute, Zagreb, Croatia; ^6^ Division of Haematology, Medical University of Graz, Graz, Austria; ^7^ Faculty of Medical Sciences, Newcastle University, Newcastle upon Tyne, United Kingdom; ^8^ Institute of Clinical Medicine, University of Oslo, Oslo, Norway; ^9^ University of Zagreb School of Medicine, Zagreb, Croatia; ^10^ Novartis Pharmaceuticals AG, Basel, Switzerland; ^11^ Department of Internal Medicine III, Hematology and Oncology, University Medical Center Mannheim, Mannheim, Germany; ^12^ Medical Clinic I, University Hospital Carl Gustav Carus, Technical University Dresden, Dresden, Germany

**Keywords:** chronic graft-versus-host disease (cGvHD), acute GvHD, aGvHD, stem cell transplantation, bone marrow transplantation

## Abstract

**Introduction:**

Chronic graft-versus-host disease (cGvHD) is a serious late complication of allogeneic hematopoietic stem cell transplantation (allo-HSCT).

**Methods:**

This multicenter analysis determined the cumulative incidence (CI) of cGvHD and late acute GvHD (laGvHD) and its impact on transplantation-related mortality (TRM), relapse (R), and overall survival (OS) in 317 patients [296 adults, 21 pediatrics (<12 years of age)] who underwent their first allo-HSCT in 2017.

**Results:**

The CI of laGvHD was 10.5% in adults and 4.8% in pediatrics, and the CI of cGvHD was 43.0% in all adult transplant patients and 50.2% in the adult at-risk cohort at the study end. The onset of cGvHD was *de novo* in 42.0% of patients, quiescent in 52.1%, and progressive in 5.9%. In adults, prophylactic use of antithymocyte globulin or posttransplant cyclophosphamide was associated with a significantly lower incidence of cGvHD (28.7%) vs. standard prophylaxis with calcineurin inhibitors (30.6%) and methotrexate/mycophenolate mofetil (58.4%) (all *p *< 0.01). TRM was significantly higher in patients with aGvHD (31.8%) vs. cGvHD (12.6%) and no GvHD (6.3%) (all *p *= 0.0001). OS in the adult at-risk cohort was significantly higher in patients with cGvHD (78.9%) vs. without (66.2%; *p *= 0.0022; HR 0.48) due to a significantly lower relapse rate (cGvHD: 14.5%; without cGvHD: 27.2%; *p *= 0.00016, HR 0.41). OS was also significantly higher in patients with mild (80.0%) and moderate (79.2%) cGvHD vs. without cGvHD (66.2%), excluding severe cGvHD (72.7%) (all *p *= 0.0214).

**Discussion:**

The negative impact of severe cGvHD on OS suggests a focus on prevention of severe forms is warranted to improve survival and quality of life.

## Introduction

Chronic graft-versus-host disease (cGvHD) has remained the most significant late complication of allogeneic hematopoietic stem cell transplantation (allo-HSCT) for the last few decades (1–6). Previous studies have shown an increasing incidence of cGvHD, ranging from 30%–70%, associated with risk factors such as advanced age of the patient, unrelated or human leukocyte antigen (HLA)–mismatched donors, and use of peripheral blood stem cells (PBSCs), but also a higher number of long-term survivors as a result of lower early non-relapse mortality (NRM) (1, 2, 4, 5, 7–14). Additionally, advanced supportive care may be associated with decreased early NRM, leading to more patients at risk of subsequent cGvHD (2, 14, 15). On the other hand, more recent studies have indicated that new prophylactic treatments, such as antithymocyte globulin (ATG) and the post-transplant administration of cyclophosphamide (PTCy), result in a lower incidence of cGvHD (16–20). Furthermore, the current National Institutes of Health (NIH) consensus criteria from 2014 for diagnosing and severity scoring of cGvHD have not been implemented in the majority of registry studies, impairing the distinction of late acute GvHD (laGvHD) from cGvHD (21). Moreover, despite its serious contribution to morbidity and mortality, there is still a limited number of studies on the incidence and outcome of laGvHD (22). Therefore, we performed a multicenter analysis of the incidence of laGvHD and cGvHD within a multicenter registry trial that included all patients transplanted at the respective centers during 2017.

## Materials and methods

This study was performed as a joint analysis within the German-Austrian-Swiss registry and expanded by centers collaborating within the European Cooperation in Science & Technology (COST) cGvHD Integrated European Network on Chronic Graft Versus Host Disease (EUROGRAFT) consortium (www.gvhd.eu), including the University Hospital Centre Zagreb (Croatia) and the University Clinical Centre Gdańsk (Poland), with the aim of identifying the accurate incidence, severity, and outcomes of chronic, classic acute, and late acute GvHD. The transplant centers of the University Hospital Regensburg (Germany), the University Hospital Dresden (Germany), the Mannheim University Hospital (Germany), the University Clinical Center Gdańsk (Poland), the University Hospital Centre Zagreb (Croatia), and the St. Anna Children's Hospital (Vienna, Austria) contributed to the analysis. The analysis included all (unselected) patients who underwent their first allo-HSCT in 2017 at the contributing transplant centers, and the endpoints were transplantation-related mortality (TRM), relapse (R), and overall survival (OS) at the last follow-up and at the second transplantation (second transplantation was censored). Disease-free survival (DFS) was assessed from transplant to the date of relapse or death from any cause. Patients were considered pediatric if they were younger than 12 years old and adults if they were 12 years or older. The adult population at risk for cGvHD contained all patients who reached day 100 after allo-HSCT without prior relapse. Detailed patient information is provided in Table 1 and is shown for the total cohort, adult patients, adult patients at risk of cGvHD, and pediatric patients (supplement) separately. To allow meaningful analysis of the incidence of cGvHD, a subset of patients at risk of developing cGvHD was selected by excluding patients with TRM or relapse before day 100 after allo-HSCT. To account for competing risks when assessing the incidence of different types of GvHD, we performed a cumulative incidence with competing risk assessment using the cmprsk package in R (20). All patients provided informed consent to share transplant details, including outcome results.

**Table 1:** Patient characteristics.

Factor	Variables	Total	Adults	Adult population at risk
		*n* = 317 (%)	*n* = 296 (%)	*n* = 249 (%)
Age group	Adult	296 (93.4)	296 (100)	249 (100)
	Children	21 (6.6)	0 (0)	0 (0)
Age median (IQR)		53 (37–61)	55 (40.5–62)	53 (39–61)
Center	Dresden	96 (32.4)	96 (32.4)	77 (30.9)
	Zagreb	69 (23.3)	69 (23.3)	59 (23.7)
	Regensburg	62 (20.9)	62 (20.9)	54 (21.7)
	Gdańsk	40 (13.5)	40 (13.5)	38 (15.3)
	Mannheim	25 (8.4)	25 (8.4)	18 (7.2)
	Vienna	4 (1.4)	4 (1.4)	3 (1.2)
Sex	Female	122 (38.5)	116 (39.2)	102 (41)
	Male	195 (61.5)	180 (60.8)	147 (59)
Main disease	Acute leukemia	182 (57.4)	174 (58.8)	147 (59)
	MDS/MPN	59 (18.6)	59 (19.9)	50 (20.1)
	Lymphoma	28 (8.8)	27 (9.1)	25 (10)
	Chronic leukemia	23 (7.3)	22 (7.4)	15 (6)
	BM failure	12 (3.8)	7 (2.4)	5 (2)
	Other	13 (4.1)	7 (2.4)	6 (2.4)
Underlying disease at transplant	1st CR	160 (50.5)	150 (50.7)	133 (53.4)
	PR, 2nd CR^a^	72 (22.7)	70 (23.6)	57 (22.9)
	>2nd CR^b^	71 (22.4)	70 (23.6)	54 (21.7)
	Other	14 (4.4)	6 (2)	5 (2)
Cell source	BM	72 (22.7)	52 (17.6)	43 (17.3)
	PBSCs	245 (77.3)	244 (82.4)	206 (82.7)
Conditioning	Non TBI, toxicity reduced	165 (52.1)	154 (52)	125 (50.2)
	Non TBI, standard dose	95 (30)	87 (29.4)	74 (29.7)
	TBI, toxicity reduced	7 (2.2)	7 (2.4)	5 (2)
	TBI, standard dose	50 (15.8)	48 (16.2)	45 (18.1)
Donor	Haploidentical	42 (13.2)	37 (12.5)	30 (12)
	HLA-matched sibling	65 (20.5)	63 (21.3)	56 (22.5)
	Unrelated	210 (66.2)	196 (66.2)	163 (65.5)
Donor gender	Female	101 (31.9)	93 (31.4)	75 (30.1)
	Male	216 (68.1)	203 (68.6)	174 (69.9)
Gender match	Female donor ➔ Male recipient	49 (15.5)	44 (14.9)	34 (13.7)
DLI	No DLI	277 (87.4)	258 (87.2)	215 (86.3)
	DLI	40 (12.6)	38 (12.8)	34 (13.7)
Prophylaxis	Standard	115 (36.3)	113 (38.2)	96 (38.6)
	ATG	135 (42.6)	129 (43.6)	111 (44.6)
	Cyclophosphamide	60 (18.9)	49 (16.6)	39 (15.7)
	Other	7 (2.2)	5 (1.7)	3 (1.2)
Days of follow-up, median (IQR)		943 (284–1,608)	1,047.5 (267–1,611)	1,388 (475–1,657)
Days of follow-up, range		0–1,962	0–1,962	100–1,962
Status at last visit^c^	2nd transplant	26 (8.2)	25 (8.4)	20 (8)
	In remission - alive	169 (53.3)	153 (51.7)	153 (61.4)
	In remission - TRM	66 (20.8)	65 (22)	39 (15.7)
	Relapse - alive	11 (3.5)	9 (3)	7 (2.8)
	Relapse - DRM	45 (14.2)	44 (14.9)	30 (12)

ATG, anti-thymocyte globulin; BM, bone marrow; CR, complete remission; DLI, donor lymphocyte infusion; DRM, disease-related mortality; HLA, human leukocyte antigen; IQR, interquartile range; MDS/MPN, myelodysplastic syndrome/myeloproliferative neoplasm; PBSC, peripheral blood stem cell; PR, partial remission; TBI, total body irradiation; TRM, transplantation-related mortality.

^a^ Or accelerated phase.

^b^ Primary refractory or blast phase.

^c^ Last visit between 2019 and 2022.

### Definitions

Acute GvHD (aGvHD) was divided into classic acute GvHD, which starts within 100 days from transplantation, and late acute GvHD, with an onset after the 100-day mark (3, 21). Additionally, laGvHD was divided into three subgroups: (1) persistent aGvHD (onset of aGvHD before day 100 with ongoing activity on day 100), (2) recurrent aGvHD (first onset of aGvHD before day 100 with resolution before day 100 and following a new episode of aGvHD after day 100), and (3) late-onset aGvHD (first onset of symptoms after day 100). Patients developing symptoms of aGvHD prior to PTCy were included in the analysis (*n* = 2).

Diagnosis and staging of cGvHD were based on its clinical manifestations according to the 2014 revised NIH criteria (3). Chronic GvHD was divided into three subgroups based on its onset: (1) progressive (direct progression of aGvHD into cGvHD or a symptom-free interval of less than 2 weeks), (2) *de novo* (cGvHD without any pre-existing aGvHD), and (3) quiescent (occurrence of cGvHD after resolved prior aGvHD) (14).

The concomitant occurrence of symptoms of aGvHD concerning the gastrointestinal tract, erythematous skin rash, and liver involvements (elevated bilirubin) was considered as an overlap syndrome subtype of cGvHD, while the hepatitis subtype of liver cGvHD with high alanine transaminase but normal bilirubin was classified as classic cGvHD.

Chronic Graft-versus-Host Disease-free and relapse-free survival (cGRFS) was defined as survival without active chronic GvHD requiring systemic treatment or disease relapse/progression at any time after transplantation as in Kawamura et al. (23).

### Statistical analysis

Descriptive statistics were used to summarize patient characteristics depending on the normality of the data. Numbers and percentages were used for categorical data, mean and standard deviation (SD) for normally distributed data, and median with interquartile range (IQR) for non-normally distributed data. Categorical variables were compared using the Chi-square test and continuous variables using the Mann-Whitney test in Medcalc (v11.4; https://www.medcalc.org/). Cumulative incidence curves for aGvHD, laGvHD, and cGvHD, as well as for disease relapse, were constructed using the cmprsk package in R (20) with TRM and relapse as competing events. The OS and DFS were assessed using the Kaplan-Meier method and the different curves were compared using a logrank test in Medcalc. *P*-values <0.05 were considered statistically significant.

## Results

### Patients

This retrospective longitudinal, observational study analyzed the data of 317 patients who underwent allo-HSCT within the year 2017 in six transplant centers (Table 1). In adults, the use of ATG (Chi-square test *p *< 0.0001) and posttransplant PTCy (*p *= 0.0005) as prophylactic agents led to a significantly lower incidence of cGvHD development compared with the standard prophylaxis with calcineurin inhibitors, cyclosporine (CSP) or tacrolimus, and methotrexate (MTX) or mycophenolate mofetil (MMF) (33.3% and 35.9% vs. 68.8%, respectively. The majority of patients with haploidentical donor (*n* = 37) received PTCy (*n* = 35) as prophylaxis, from which 28.6% (*n* = 10) developed cGvHD. Patients with identical siblings as donors (*n* = 63) who had ATG (*n* = 15) as prophylaxis developed cGvHD in 13.3% (*n* = 2), while those who received PTCy (*n* = 2) did not develop cGvHD. Standard prophylaxis (*n* = 45) led to 57.8% (*n* = 26) cGvHD patients. In patients with unrelated donors (*n* = 196) the protective effect of ATG (*n* = 114) and PTCy (*n* = 13) for cGvHD was also confirmed compared to standard (*n* = 66) prophylaxis (30.7%, *n* = 35 and 46.2%, *n* = 6 vs. 59.1%, *n* = 39 respectively) taking into account that nearly all patients with one exception receiving PTCy were grafted with peripheral stem cells. In total, cGvHD incidence was lowest in haploidentical patients (27.0% vs. identical 44.4% and unrelated 41.8%, Supplementary Table S1). Furthermore, cGvHD development was associated with the use of PBSCs (*p *= 0.0004), whereas previous aGvHD and laGvHD did not show a significant association with subsequent cGvHD (*p *= 0.0708) due to a significant proportion of *de novo* cGvHD.

### Characteristics of aGvHD, laGvHD, and cGvHD

Due to the significant differences in the adult and pediatric populations, the results of the adult population are presented here, and the details of the pediatric patients are provided in the Supplementary Material.

Throughout the observation time of all adult patients, with a median of 1,047 (range 0–1,962) days, aGvHD occurred in 128 (43.2%) adult patients, starting at a median of 32 (range 1–93) days, with a median overall grade at onset of 2 (range 1–4) (Table 2). Detailed organ stages are shown in Supplementary Figure S1. The maximum severity of aGvHD was reached at a median of 36 (range 4–100) days after transplantation. Steroid-refractory acute GvHD was reported in 21 (16.4%) adults.

**Table 2:** Characteristics of acute and late acute gvHD.

		Total	Adults	Adult population at risk
		*n* = 317 (%)	*n* = 296 (%)	*n* = 249 (%)
aGvHD	aGvHD	134 (42.3)	128 (43.2)	119 (47.8)
	No aGvHD	183 (57.7)	168 (56.8)	130 (52.2)
Grade of aGvHD at onset	1	62 (46.3)	57 (44.5)	54 (45.4)
	2	56 (41.8)	55 (43)	54 (45.4)
	3	12 (9)	12 (9.4)	8 (6.7)
	4	4 (3)	4 (3.1)	3 (2.5)
Grade of aGvHD at max	1	43 (32.1)	39 (30.5)	37 (31.1)
	2	65 (48.5)	64 (50)	62 (52.1)
	3	17 (12.7)	16 (12.5)	14 (11.8)
	4	9 (6.7)	9 (7)	6 (5)
Steroid-refractory aGvHD		25 (18.7)	21 (16.4)	18 (15.1)
laGvHD	laGvHD	32 (10.1)	31 (10.5)	30 (12)
	No laGvHD	285 (89.9)	265 (89.5)	219 (88)
laGvHD onset	Late onset *de novo*	16 (50)	16 (51.6)	15 (50)
	Persistent	9 (28.1)	8 (25.8)	8 (26.7)
	Recurrent	7 (21.9)	7 (22.6)	7 (23.3)
Grade of laGvHD severity at onset	1	7 (21.9)	7 (22.6)	7 (23.3)
	2	12 (37.5)	12 (38.7)	11 (36.7)
	3	10 (31.3)	9 (29)	9 (30)
	4	3 (9.4)	3 (9.7)	3 (10)
Grade of laGvHD at maximum severity	1	6 (18.8)	6 (19.4)	6 (20)
	2	8 (25)	8 (25.8)	7 (23.3)
	3	11 (34.4)	11 (35.5)	11 (36.7)
	4	7 (21.9)	6 (19.4)	6 (20)
Steroid-refractory laGvHD		8 (25)	7 (22.6)	6 (20)
Steroid-sensitive laGvHD		24 (75)	24 (77.4)	24 (80)

aGvHD, acute graft-versus-host disease; laGvHD, late aGvHD.

The laGvHD started in 31 (10.5%) adult patients at a median of 138 days after transplantation (range 100–525 days), with a median grade at onset of 2 (range 1–4) (Table 2). The maximum severity was reached at a median of 167 (range 100–541) days after transplantation, with a median grade of 3 (range 1–4) (Table 2). In adult patients, persistent laGvHD was observed in 8 (25.8%) patients and recurrent laGvHD in 7 (22.6%) patients, while late-onset laGvHD was seen in 16 (51.6%) patients. A summary of organ grades is shown in Supplementary Figure S2 and detail in Supplementary Table S2. Steroid-refractory laGvHD was reported in seven (22.6%) patients.

Based on the complete cohort of 317 patients, a total of 122 (38.5%) patients developed cGvHD: 120 (40.5%) adults and 2 (9.5%) pediatrics (Table 3). For the calculation of cGvHD incidence, we excluded 47 adult patients with TRM (*n* = 26, 8.8%), relapse (*n* = 18, 6.1%), or second transplantation (*n* = 3, 1.0%) before day 100, resulting in 249 adult patients at risk (Supplementary Table S3). Out of this cohort, 47.8% (*n* = 119) were diagnosed with cGvHD according to the 2014 revised NIH consensus criteria, at a median time of 201 (range 68–1,051) days. cGvHD severity at onset was mild in 60 (50.4%) patients, moderate in 48 (40.3%) patients, and severe in 11 (9.2%) patients (Table 3). The maximum severity was reached at a median of 236 (range 68–1,129) days after clinical onset of cGvHD; cGvHD severity at maximum was mild in 53 (44.5%) patients, moderate in 45 (37.8%) patients, and severe in 21 (17.6%) patients.

**Table 3:** Cgvhd characteristics.

Total		Total	Adults	Adult population at risk
		*n* = 317 (%)	*n* = 296 (%)	*n* = 249 (%)
cGvHD	cGvHD	122 (38.5)	120 (40.5)	119 (47.8)
	No cGvHD	195 (61.5)	176 (59.5)	130 (52.2)
cGvHD type of onset	De novo	51 (41.8)	51 (42.5)	50 (42)
	Quiescent	63 (51.6)	62 (51.7)	62 (52.1)
	Progressive	8 (6.6)	7 (5.8)	7 (5.9)
cGvHD classification	Classic	101 (82.8)	100 (83.3)	99 (83.2)
	Overlap	21 (17.2)	20 (16.7)	20 (16.8)
Grade of cGvHD severity at onset	Mild	60 (49.2)	60 (50)	60 (50.4)
	Moderate	50 (41)	49 (40.8)	48 (40.3)
	Severe	12 (9.8)	11 (9.2)	11 (9.2)
Days from tx to cGvHD max symptoms, median (IQR)		241 (168–378)	241 (167–385)	236 (167–389)
Days from tx to cGvHD max symptoms, range		68–1,129	68–1,129	68–1,129
Platelets <100/nl at onset		18 (14.8)	17 (14.2)	17 (14.3)
Second-line therapy required		35 (28.7)	34 (28.3)	34 (28.6)
Systemic immunosuppression at onset of cGvHD	Yes	79 (64.8)	78 (65)	78 (65.5)
	No	43 (35.2)	42 (35)	41 (43.5)

cGvHD, chronic graft-versus-host disease; IQR, interquartile range; Tx, transplantation.

Most adults at risk had quiescent (*n* = 62, 52.1%) cGvHD with previously resolved aGvHD, followed by *de novo* cGvHD (*n* = 50, 42.0%) and progressive (*n* = 7, 5.9%) cGvHD. The majority of adult patients showed classical manifestation (*n* = 99, 83.2%), whereas overlap syndrome was diagnosed in 16.8% (*n* = 20) of cases. Patients who developed cGvHD within 100 days were not excluded. Nine patients were diagnosed with cGvHD (44.4% *de novo*, 55.6% quiescent) before day 100, with seven showing only manifestations of cGvHD (classic form) and two having overlap syndrome.

The most frequently affected organs were oral mucosa (52.1%), skin (40.3%), eyes (26.1%), and liver (25.2%), followed by gastrointestinal tract (12.6%), joints and fascia (5.9%), lungs (5.0%), and genital tract (3.7%). Mild cGvHD was mostly caused by oral (50.0%), skin (30.0%), and liver (15.0%) manifestations. Moderate cGvHD was dominated by oral (56.3%), skin (47.9%), eye (39.6%), and liver (35.4%) manifestations. Severe cGvHD mainly included skin (63.6%), oral (45.5%), eye (36.6%), and liver (36.6%) manifestations (Supplementary Figure S3). The median number of organs involved at the time of onset was one (IQR 1–2, range 1–5) in adults at risk. Detailed organ stages are shown in Supplementary Tables S4, S5. Three (2.5%) patients were diagnosed with cGvHD with isolated associated manifestations (glomerulonephritis and cerebral vasculitis).

Progression to cGvHD was observed in 11 of the 30 adult patients at risk with laGvHD (36.7%, Tables 4, 5). A total of 34 (13.7%) adults at risk received a donor lymphocyte infusion; however, 29.4% of them developed cGvHD (details in Supplementary Table S6). Risk factors for the development of cGvHD are shown in Table 6.

**Table 4:** Lagvhd progressing to cGvHD.

laGvHD	cGvHD	cGvHD onset type	Adult population at risk	Adults total
			*n* = 249 (%)	*n* = 296 (%)
No laGvHD			219 (88)	265 (89.5)
	No cGvHD		111 (44.6)	156 (52.7)
	cGvHD		108 (43.4)	109 (36.8)
		De novo	50 (46.2)	51 (46.8)
		Progressive	2 (1.9)	2 (1.8)
		Quiescent	56 (51.9)	56 (51.4)
laGvHD			30 (12)	31 (10.5)
	No cGvHD		19 (7.6)	20 (6.8)
	cGvHD		11 (4.4)	11 (3.7)
		De novo	0 (0)	0 (0)
		Progressive	5 (45.5)	5 (45.5)
		Quiescent	6 (54.5)	6 (54.5)

cGvHD, chronic graft-versus-host disease; laGvHD, late acute gvHD.

**Table 5:** laGvHD development dependent on cGvHD onset type.

cGvHD	cGvHD onset	laGvHD	Adult population at risk	Adults total
			*n* = 249 (%)	*n* = 296 (%)
No cGvHD			130 (52.2)	176 (59.5)
cGvHD			119 (47.8)	120 (40.5)
	De novo		50 (42.0)	51 (42.5)
	Progressive		7 (5.9)	7 (5.8)
		No laGvHD	2 (28.6)	2 (28.6)
		laGvHD	5 (71.4)	5 (71.4)
	Quiescent		62 (52.1)	62 (51.7)
		No laGvHD	56 (90.3)	56 (90.3)
		Lagvhd	6 (9.7)	6 (9.7)

cGvHD, chronic graft-versus-host disease; laGvHD, late acute gvHD.

**Table 6:** Risk factors for subsequent cGvHD in adult population at risk.

		cGvHD	No cGvHD	*P*-value
		*n* = 119 (%)	*n* = 130 (%)	
Age	Mean (range)	51.3 (20–74)	48 (12–71)	0.1666
	Median (IQR)	53 (43–62)	51.5 (38–61)	
Sex	Male	68 (46.3)	79 (53.7)	0.5619
	Female	51 (50)	51 (50)	
Main disease	Acute leukemia	73 (49.3)	75 (50.7)	0.4984
	MDS/MPN	23 (46)	27 (54)	
	Lymphoma	9 (36)	16 (64)	
	Chronic leukemia	10 (66.7)	5 (33.3)	
	BM failure	2 (40)	3 (60)	
	Other	2 (33.3)	4 (66.7)	
Remission status at tx	CR	59 (44.4)	74 (55.6)	0.4846
	PR, 2nd CR, or accelerated phase	27 (47.4)	30 (52.6)	
	No remission	30 (55.6)	24 (44.4)	
	Other	3 (60)	2 (40)	
Donor sex	Male	91 (52.3)	83 (47.7)	0.0304
	Female	28 (37.3)	47 (62.7)	
Donor sex match	Female donor ➔ Male recipient	10 (29.4)	24 (70.6)	0.0941
Donor type	Haplo	10 (33.3)	20 (66.7)	0.2394
	Identical related	28 (50)	28 (50)	
	Unrelated	81 (49.7)	82 (50.3)	
Cell source	BM	10 (23.3)	33 (76.7)	0.0004
	PBSCs	109 (52.9)	97 (47.1)	
Prophylaxis	Standard	66 (68.8)	30 (31.3)	
	ATG	37 (33.3)	74 (66.7)	<0.0001*
	PTCy	14 (35.9)	25 (64.1)	0.0005*
	Other	2 (66.7)	1 (33.3)	0.9392*
DLI	DLI	10 (29.4)	24 (70.6)	0.0212
	No DLI	109 (50.7)	106 (49.3)	
TBI	TBI	27 (54)	23 (46)	0.3265
	No TBI	92 (46.2)	107 (53.8)	
aGvHD	aGvHD	64 (53.8)	55 (46.2)	0.0708
	No aGvHD	55 (42.3)	75 (57.7)	
	Grade 0–2	114 (47.9)	124 (52.1)	0.8742
	Grade 3–4	5 (45.5)	6 (54.5)	
	Early or late acute	69 (51.1)	66 (48.9)	0.2547
	No early or late acute	50 (43.9)	64 (56.1)	

ATG, anti-thymocyte globulin; BM, bone marrow; CR, complete remission; IQR, interquartile range; MDS/MPN, myelodysplastic syndrome/myeloproliferative neoplasm; PBSC, peripheral blood stem cell; PR, partial response; PTCy, posttransplant cyclophosphamide; TBI, total body irradiation.

* Compared with standard prophylaxis.

### Cumulative incidence

**Figure 1:**
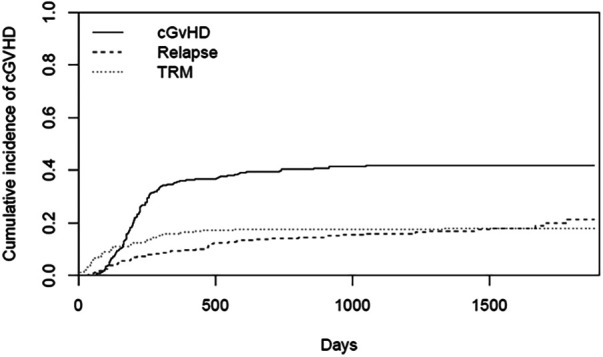
CI of cGvHD in all adults (*n* = 296). cGvHD, chronic graft-versus-host disease; CI, cumulative incidence; TRM, transplantation-related mortality.

**Figure 2:**
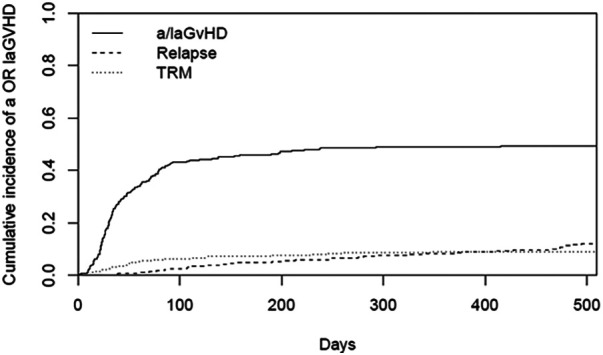
CI of combined aGvHD and laGvHD in all adults (*n* = 296). aGvHD, acute GvHD; CI, cumulative incidence; GvHD, graft-versus-host disease; laGvHD, late acute GvHD; TRM, treatment-related mortality.

The cumulative incidence of aGvHD (at day 100), laGvHD (at day 365), and cGvHD (at day 365 and at the end of study) was 43.4%, 10.5%, 36.3%, and 43.0%, respectively, (Figure 1) including all adult patients. The combined incidence of aGvHD and laGvHD was 49.5% at day 365 and 49.6% at the end of the study (Figure 2). When focusing on the adult patients at risk, the cumulative incidence of cGvHD was 42.3% at day 365 and 50.2% at the end of the study (Supplementary Figure S4).

### Outcome of laGvHD

Due to the low number of patients (*n* = 31) statistical analyses failed to detect any significant effects. However, TRM and Relapse in the total adult population were both higher in patients developing laGvHD compared to patients without laGvHD (66.5% vs. 76.0% and 50.2% vs. 70.3%, Supplementary Figure S5). In addition, patients with laGvHD had a minimal worse outcome in OS compared to patients without laGvHD (56.1% vs. 60.2%, Supplementary Figure S5).

### Treatment of cGvHD

At the onset of cGvHD, 65.5% of the adult patients at risk continued to receive systemic immunosuppression. Twelve patients (10.1%) remained initially untreated, while most patients (*n* = 107, 89.2%) received intensified immunosuppressive therapy by either an increase in the dosage of the GvHD prophylaxis (*n* = 14, 11.8%), or the start of first-line therapy (*n* = 93, 78.2%) with a single agent (*n* = 33, 35.5%), or a combination of two (*n* = 53, 57.0%) or three (*n* = 7, 7.5%) agents. Due to insufficient response to the first-line therapy, 34 (28.6%) of the 119 patients with cGvHD required second-line therapy. Two (14.3%) of the 14 patients receiving an increased dosage of prophylaxis as first-line treatment only required second-line therapy, while 33.3% (*n* = 11), 34.0% (*n* = 18), and 42.9% (*n* = 3) of the patients receiving single-, double-, or triple-agent therapy needed second-line treatment after a median of 149 days (5 months), respectively (Table 7).

**Table 7:** cGvHD treatment and need of second-line therapy in adults at risk.

cGvHD treatment	Total	Second-line therapy	
		Needed	Not needed
	*n* = 119 (%)	*n* = 34 (%)	*n* = 85 (%)
No systemic immunosuppresion	12 (10.1)	0 (0)	12 (100.0)
Increase of prophylaxis only	14 (11.8)	2 (14.3)	12 (85.7)
Single agent	33 (27.7)	11 (33.3)	22 (66.7)
Double agent	53 (44.5)	18 (34.0)	35 (66.0)
Triple agent	7 (5.9)	3 (42.9)	4 (57.1)

Cgvhd, chronic graft-versus-host disease.

### First-line therapy

First-line monotherapy was administered in 33 (27.7%) adult patients at risk, mainly using steroids (90.9%); MTX was used in 2 (6.1%) patients and CSP in one (3.0%) patient. Double-agent therapy was used in 53 (44.5%) patients, consisting mostly of the combination of steroids and CSP (*n* = 30, 56.6%), followed by steroids and tacrolimus (*n* = 11, 20.8%), and steroids and MTX (*n* = 3, 5.7%).

Seven (5.0%) adult patients received upfront triple-agent therapy with combinations of steroids, tacrolimus, and MMF, or steroid, CSP, and extracorporeal photopheresis. Detailed information on the regimens is shown in Supplementary Tables S7–S9.

Of the 30 adult patients who exclusively received steroids for initial treatment, six (20.0%) had a complete response after 3 months, two (6.7%) had a mixed response, three (10.0%) had stable disease, seven (23.3%) had a partial response, and three (10.0%) had progression of cGvHD, while in two (6.7%) patients follow-up was lacking. In seven patients, the steroid therapy lasted less than 3 months, as five (16.7%) patients were switched to other therapies or had the steroids stopped because cGvHD was resolved, and two (6.7%) patients died. After 6 months of steroid-only therapy, seven (23.3%) patients had a complete response, one (3.3%) had a mixed response, three (10.0%) had a partial response, three (10.0%) had stable disease, and four (13.3%) had further progression (Table 8).

**Table 8:** Three- and six-month responses to steroids only in first-line therapy in adult cGvHD patients (*n* = 30).

Response	Response to therapy at	
	3 months	6 months
Complete response	6 (20%)	7 (23.3%)
Partial response	7 (23.3%)	3 (10%)
Mixed response	2 (6.7%)	1 (3.3%)
Stable disease	3 (10%)	3 (10%)
Progression	3 (10%)	4 (13.3%)
Therapy prematurely terminated	5 (16.7%)	7 (23.3%)
Died on therapy	2 (6.7%)	3 (10%)
Lost to follow-up	2 (6.7%)	2 (6.7%)

cGvHD, chronic graft-versus-host disease.

### Second-line therapy

Second-line treatment was administered to 34 (28.6%) adult patients with cGvHD, starting at a median time of 149.5 (IQR 48–307, range 9–658) days after the onset of cGvHD.

At the start of second-line therapy, four (11.8%) patients had mild cGvHD, 15 (44.1%) had moderate cGvHD, and 15 (44.1%) had severe cGvHD. Only 11 (18.3%) of 60 patients with initially mild cGvHD required second-line therapy. In contrast, 18 (37.5%) of the 48 initially moderate cGvHD cases and five (45.5%) of 11 patients with initially severe cGvHD required subsequent second-line treatment (see Supplementary Figures S6, S7).

### Outcome with second-line therapy

A total of 34 (28.6%) of all adult patients with cGvHD received second-line therapy; 29 (85.3%) of those were alive at last contact, two (5.9%) patients were lost to follow-up, and five (14.7%) patients died due to TRM or relapse (Supplementary Table S10).

### Outcome of cGvHD

Of the 249 patients reaching day 100, 129 (51.8%) were alive in remission at the last follow-up. Overall outcomes after first- and/or second-line therapy in the adult at-risk patients are shown in Table 9. The main outcomes of interest were TRM, relapse, and OS at the last follow-up. Patients undergoing a second transplant were excluded from subsequent analyses regardless of when the second transplant was performed and were censored for survival analyses (*n* = 20).

**Table 9:** Overall outcomes in the adult population at risk (*n* = 249) according to the cGvHD status and treatment.

		No cGvHD	cGvHD			Total
			0 or 1st line	2nd line	Total cGvHD	
		*n* = 130 (%)	*n* = 85 (%)	*n* = 34 (%)	*n* = 119 (%)	*n* = 249 (%)
Remission	Alive	54 (41.5)	48 (56.5)	27 (79.4)	75 (63)	129 (51.8)
	TRM	25 (19.2)	10 (11.8)	4 (11.8)	14 (11.8)	39 (15.7)
Relapse		23 (17.7)	13 (15.3)	1 (2.9)	14 (11.8)	37 (14.9)
	DRM	19 (14.6)	10 (11.8)	1 (2.9)	11 (9.2)	30 (12)
	Alive	4 (3.1)	3 (3.5)	0 (0)	3 (2.5)	7 (2.8)
2nd transplant		16 (12.3)	4 (4.7)	0 (0)	4 (3.4)	20 (8)
Lost to follow-up		12 (9.2)	10 (11.8)	2 (5.9)	12 (10.1)	24 (9.6)

cGvHD, chronic graft-versus-host disease; DRM, disease-related mortality; TRM, transplantation-related mortality.

### Transplantation-related mortality

**Figure 3:**
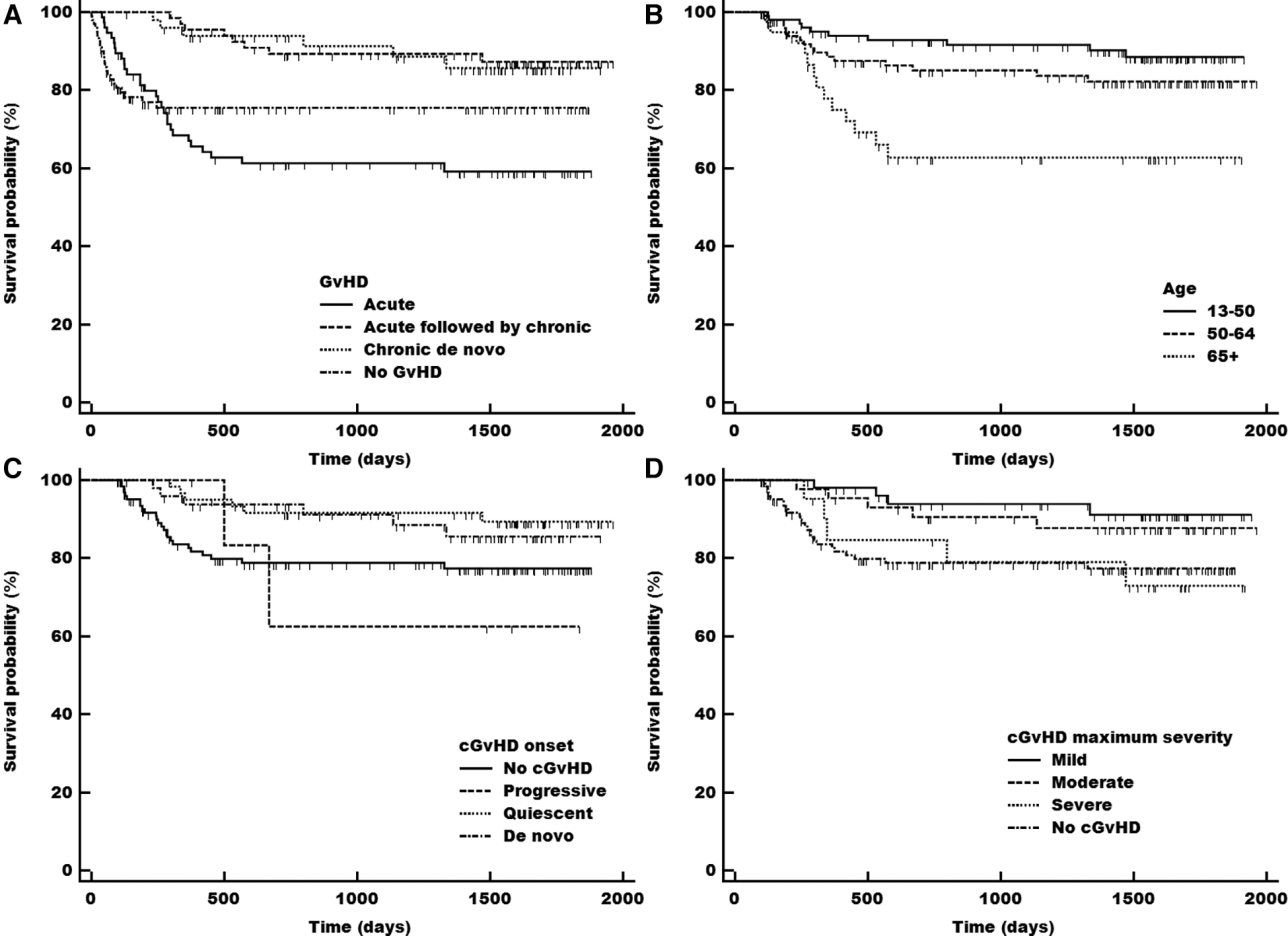
Transplant related mortality (TRM) in total adult population according to the acute (early and late), chronic (including previous acute) and no gvDH status (**A**), and in population at risk according to patients’ age (**B**), onset type of cGvHD (**C**), maximum severity grade of cGvHD (**D**) cGvHD, chronic gvHD; gvHD, graft-versus host disease.

When the 249 patients at risk of cGvHD or laGvHD were divided into subgroups of “aGvHD only”, “aGvHD followed by cGvHD”, “no GvHD”, and “cGvHD only”, we found significantly higher TRM in patients with aGvHD compared with patients with cGvHD and no GvHD (31.8% vs. 12.6% and 6.3%, respectively; *p *= 0.0001, Figure 3A). TRM was significantly higher in elderly patients above 65 years (31.7%) compared with that in patients between 50 and 64 years (17.0%) of age and in patients between 13 and 50 years of age (9.3%) (all *p *= 0.0009, Figure 3B). Although we did not find a significant influence of onset type (*p *= 0.1224, Figure 3C) or onset severity of cGvHD (*p *= 0.0937, Supplementary Figure S8B) on TRM, progressive onset appeared to have the worst outcome but performed better compared with historical cohorts (1, 23). TRM was significantly lower in mild and moderate maximum-severity cGvHD compared with severe and no cGvHD (7.6% and 11.1% vs. 23.8% and 19.2%, respectively; *p *= 0.0478, Figure 3D). Through multivariate analysis we did not find significant influence on TRM.

### Disease-free survival

**Figure 4:**
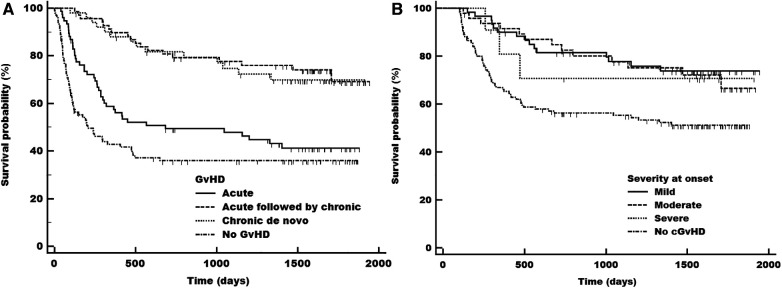
Disease-free survival (DFS) in total adult population at risk according to the acute (early and late), chronic (including previous acute) and no gvDH status (**A**) and in population at risk according to severity grade at onset of cGvHD (**B**) cGvHD, chronic graft-versus-host disease.

DFS was significantly higher in patients with cGvHD (73.9%) and independent of previous aGvHD (72.6%) compared with aGvHD only (43.4%) and no GvHD at all (39.0%) (all *p *< 0.0001, Figure 4A). The worst DFS was seen in patients without any GvHD, in part due to transplantation-related death events within the first 100 days after transplantation, but these patients showed the most stable long-term survival when reaching the 500-day mark. Although the appearance of aGvHD resulted in a similar impaired survival probability, the combination of aGvHD and cGvHD acted as a beneficial factor on DFS. The severity of cGvHD at onset did not impact DFS (mild: 75%, moderate: 72.9%, and severe: 72.7%), but compared with no cGvHD, all severity grades had a statistically significant better outcome in DFS (*p *= 0.0016, Figure 4B).

### Relapse

The cumulative incidence of relapse for patients with and without cGvHD was statistically different (*p *< 0.0001; Supplementary Figure S9A), with cGvHD patients having a lower relapse incidence compared with patients without cGvHD (15.2% vs. 32.9%). The type of cGvHD onset (*p *= 0.4566) or severity (*p *= 0.8110) did not impact the relapse incidence (Supplementary Figure S9B). The cumulative incidence of relapse with TRM as a competing risk was 25.5% at the end of the study. The multivariate analysis showed aGvHD as a protective factor for relapse [HR 0.5 (0.3–0.9); *p* = 0.0155].

### Overall survival

The OS was significantly higher in patients with cGvHD compared with patients without cGvHD in the adult at-risk population [79.0% vs. 66.2%; logrank test *p *= 0.0022; hazard ratio 0.35, 95% confidence interval (CI) 0.3–0.77]. Patients with aGvHD had similar outcomes to patients without any form of GvHD (50% vs. 55%; logrank test *p *< 0.0001; Supplementary Figure S10A).

In terms of the different grades of cGvHD at onset, there was a significant difference in the OS of patients with mild and moderate cGvHD compared with patients without cGvHD, but not for severe cGvHD (mild: 80.0%; moderate: 79.2%; severe: 72.7% vs. no cGvHD: 66.2%; logrank test *p *= 0.0214; Supplementary Figure S10B). However, there were no statistically significant differences between the individual severity grades when limited to cGvHD patients only (*p *= 0.7396).

Also, when limited to at-risk cGvHD patients only, we found a significant influence of cGvHD classification at the time of evaluation, with overlap symptoms carrying a 4.8 times higher risk of death (95% CI 1.5–15.1; logrank test *p *= 0.0071; Supplementary Figure S10C), but no significant influence of onset type of cGvHD on OS (*p *= 0.1282). Both analyses were limited by the number of cases with overlap or progressive cases.

In the total adult population (*n* = 296), there was a significantly higher survival with bone marrow (BM) as stem cell source (*p *= 0.0211; Supplementary Figure S10D), but no significant effect of aGvHD (*p *= 0.0583), laGvHD (*p* = 0.8652), donor sex (*p *= 0.6525), intensity of conditioning (*p *= 0.1314), total body irradiation conditioning (*p *= 0.1415), or combinations thereof (*p *= 0.0572) on the OS.

On the other hand, the OS was not significantly affected by the underlying disease at the time of transplant (*p *= 0.0550) or cGvHD prophylaxis (*p *= 0.3017). Additional details are shown in the Supplementary Figure S1^1^.

In multivariate analysis PBSC stem cell source [HR 3.3 (1.3–8.5); *p* = 0.0145] was associated with reduced overall survival. Patients' age also showed significant influence on overall survival in multivariate analysis [HR 1.0 (1.01–1.05); *p* = 0.0078].

### Graft-versus-Host disease-free and relapse-free survival (GRFS)

**Figure 5:**
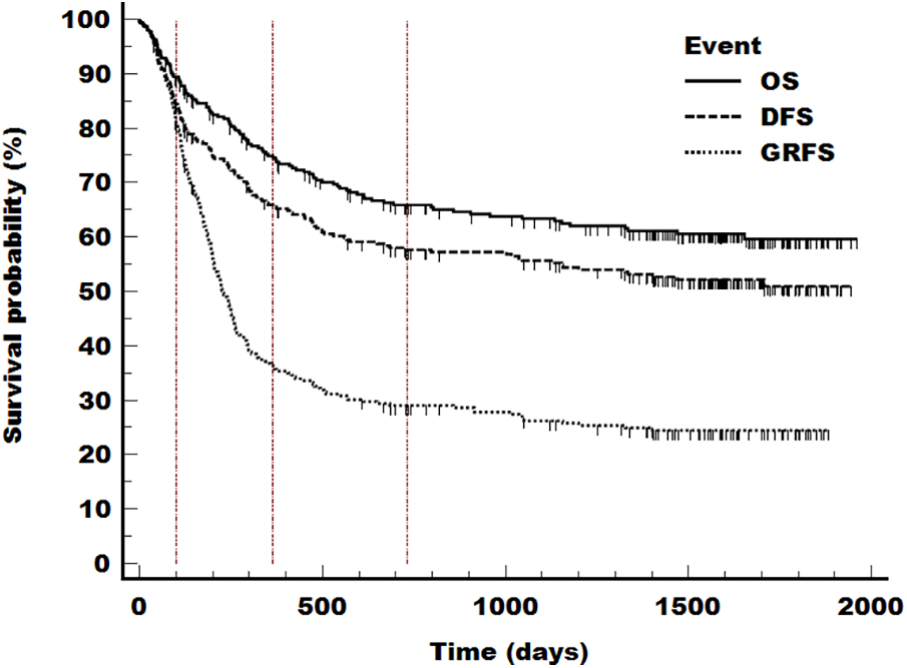
GRFS, OS and DFS. OS, overall survival; DFS, disease-free survival; GRFS, Graft-versus-Host Disease-free and relapse-free survival. The Kaplan Meier curve depicts the following endpoints relapse, death and need for systemic treatment of chronic GvHD. All events were counted equally.

Chronic GRFS at day 100 and day 365 was 81% and 36.1%. The total GRFS at end of follow up was 24.4% (Figure 5).

### Landmark analysis

The landmark analysis of the population alive at day 100 (excluding patients who died prior to day 100) equals our population at risk taking into account that patients with relapse were also excluded (*n* = 13), which did not affect the survival curves significantly. Compared to the complete adult population from day 0, we found a better outcome for patients without any GvHD in the day 100 landmark analysis. The outcome of patients with aGvHD and cGvHD remained unaltered (Figures 3A, 4A, Supplementary Figures S10A vs. S1^2^).

## Discussion

The aim of this study was to identify the correct incidence and outcome of cGvHD and laGvHD in a prospective multicenter analysis including all transplanted patients during the year 2017 at multiple centers in Europe.

Most importantly, the analysis revealed for the first time a lower incidence of cGvHD compared with published data (5, 24, 25) within an unselected patient cohort included at the day of transplant to provide the accurate incidence within a real-world population. To eliminate survivorship bias, we focused on the population at risk, demonstrating that patients without any kind of GvHD had a better survival outcome. Nevertheless, in both cohorts (patients at risk vs. all patients), aGvHD was associated with the worst outcome in OS, while patients with cGvHD still had the best OS rate. Multiple risk factors for the development of cGvHD have been identified in the last few years, such as the age of the patient, use of unrelated or HLA-mismatched donors, and use of PBSCs (1, 2, 4, 5, 7–13). In agreement with published data (25), we detected a correlation between cGvHD and the age of the patients as well as use of PBSCs, but contrary to prior publications (24), no significant influence of previous aGvHD and laGvHD on the development of cGvHD. The latter can be explained by several factors including significant heterogeneity of patient characteristics combined with a medium sized total population, a relatively high median age of patients who in addition had a high risk of relapse leading to early termination of immunosuppression which led to a relatively high proportion of *de novo* cGvHD (42.5%). Our study also confirmed the use of ATG and PTCy for GvHD prophylaxis as protective factors, which is consistent with previous reports (17, 19). Interestingly, the type of applied GvHD prophylaxis had a significant effect on cGVHD incidence while HLA-matching had no detectable effect on incidence of cGVHD with a cGvHD rate of 15%–30% in patients with use of ATG and 30%–45% in patients with PTCy as prophylaxis independent on the donor type and HLA-match while standard prophylaxis led to 60% cGvHD development in related and unrelated donor transplant.

In our study, nearly half of the patients developed quiescent onset of cGvHD after resolution of aGvHD, which is in line with published data (26). At the onset of cGvHD, nearly 70% of patients were still receiving systemic immunosuppression. Not surprisingly, diagnosis of moderate or severe cGvHD resulted in a higher incidence of subsequent second-line therapy compared with patients with mild cGvHD. As was already known from previous studies, we were able to confirm the significant influence of aGvHD and severe cGvHD on TRM in comparison with patients without cGvHD (24, 27).

Nearly one-third of the adult patients with cGvHD were treated with steroids only. After 3 months, 20% had progression and 20% had complete remission (CR); after 6 months, CR increased marginally, while the progression rate was consistent. In total, 28.6% of all adult patients with cGvHD were in need of second-line therapy, of which 85.3% survived, which is higher than the INTEGRATE trial and comparable to the GRAVITAS-309 trial, taking into account the significant patient heterogeneity of the disease (28, 29). Steroid-refractory aGvHD was associated with a significantly lower OS than steroid-sensitive aGvHD.

Concordant with the existing data, OS was significantly higher in patients with cGvHD compared with patients without cGvHD, as the diagnosis of cGvHD resulted in a significantly lower relapse rate (24, 30, 31), while severity or type of onset of cGvHD had no impact on R or TRM. Of note, our cohort revealed a higher survival rate, not only in mild but also in moderate cGvHD, compared with that in patients without cGvHD, most likely due to the associated graft-versus-leukemia effect reducing the relapse rate as reported in previous studies and decreased TRM in the latter population (31). In contrast, severe cGvHD continued to negatively affect OS, and future studies should focus on the prevention of severe forms to improve OS and quality of life. In addition, relapse continues to impact OS and additional strategies are required to reduce relapse-related mortality. We also found an improved long-term survival in patients with BM as stem cell source compared with PBSCs, which is in line with published data (32). Treatment of cGvHD with a triple-agent regimen was associated with a significantly lower OS due to its use predominantly in severe cGvHD.

Our study has some limitations. Due to the multicenter analysis, a variety of conditioning regimens and GvHD prophylaxis were used, which may have had an impact on the incidence of GvHD. Furthermore, we restricted our observation to patients who had been transplanted within one year, which limited the size of our patient collective. Additionally, the pediatric cohort was too small for a meaningful analysis of the influencing factors of cGvHD.

In future, larger datasets applying the NIH criteria of cGvHD are needed to confirm the cumulative incidence and outcome of GvHD, especially in pediatric patients.

## Data Availability

The raw data supporting the conclusions of this article will be made available by the authors, without undue reservation.
