# Supplementary material for: Retrospective analysis of the incidence and outcome of late acute and chronic graft-versus-host disease—an analysis from transplant centers across Europe
Source: Front Transplant. 2024 Mar 18;3:1332181. doi: 10.3389/frtra.2024.1332181 (PMC11235324; doi:10.3389/frtra.2024.1332181)
Supplement: Supplementary file 1 [file Datasheet1.pdf]

Supplementary Material

Retrospective analysis of the incidence and outcome of late acute and chronic graft-versus-host disease - an analysis from transplant centers across Europe

Ronja Langer[1], Antonela Lelas[2], Michael Rittenschober[3], Agnieszka Piekarska[4], Alicja Sadowska-Klasa[4], Ivan Sabol[5], Lana Desnica[2], Hildegard Greinix[6], Anne Dickinson[7], Marit Inngjerdingen[8], Anita Lawitschka[3,9], Radovan Vrhovac[2,10], Drazen Pulanic[2,10], Sibel Güneş[11], Stefan Klein[12], Jan Moritz Middeke[13], Matthias Grube[1], Matthias Edinger[1], Wolfgang Herr[1], and Daniel Wolff[1*]

*** Correspondence:**
Daniel Wolff, MD
Department of Internal Medicine III, Hematology and Oncology
University Hospital Regensburg
Franz-Josef Strauß Allee 11
93053 Regensburg
Germany
E-mail: daniel.wolff@ukr.de
Phone: +49-941-944-15542
Fax: +49-941-944-5543

Pediatric analysis

In children, aGvHD started at a median of 24.5 (range 17–42) days, with a median overall stage at onset of 1 (range 1–2). The maximum severity occurred at a median of 38.5 (range 24–97) days after transplantation with a median stage of 1 (range 1–3).

Steroid-refractory aGvHD occurred in four (66.7%) pediatric patients. One pediatric patient developed steroid-refractory, persistent late acute GvHD (laGvHD) at day 100 with onset stage 3, and reached maximum severity stage 4 at day 125. Two patients were excluded due to death and relapse within 100 days, leading to a cohort of 19 pediatric patients at risk. Of those, two children (10.5%) developed cGvHD at a median time of 208.5 (range 196–221) days, both with moderate severity. The number of organs involved at time of onset was four and six in the two pediatric patients, respectively.

Only two pediatric patients required second-line therapy, but it was administered to one patient 77 days after diagnosis.

Table A Pediatric patient characteristics

Factor	Variables	Children n=21 (%)
Age median (IQR)		4 (1–5.5)
Center	Dresden	0 (0)
	Zagreb	6 (28.6)
	Regensburg	0 (0)
	Gdańsk	0 (0)
	Mannheim	0 (0)
	Vienna	15 (71.4)
Sex	Female	6 (28.6)
	Male	15 (71.4)
Main disease	Acute leukemia	8 (38.1)
	MDS/MPN	0 (0)
	Lymphoma	1 (4.8)
	Chronic leukemia	1 (4.8)
	BM failure	5 (23.8)
	Other	6 (28.6)
Underlying disease at transplant	1st CR	10 (47.6)
	PR, 2nd CR*	2 (9.5)
	>2nd CR**	1 (4.8)
	Other	8 (38.1)
Cell source	BM	20 (95.2)
	PBSCs	1 (4.8)
Conditioning	Non TBI, toxicity reduced	11 (52.4)
	Non TBI, standard dose	8 (38.1)
	TBI, toxicity reduced	(0)
	TBI, standard dose	2 (9.5)
Donor	Haploidentical	5 (23.8)
	HLA-matched sibling	2 (9.5)
	Unrelated	14 (66.7)
Donor gender	Female	8 (38.1)
	Male	13 (61.9)
Gender match	Female donor → Male recipient	5 (23.8)
DLI	No DLI	19 (90.5)
	DLI	2 (9.5)
Prophylaxis	Standard	2 (9.5)
	ATG	6 (28.6)
	Cyclophosphamide	11 (52.4)
	Other	2 (9.5)
Days of follow-up, median (IQR)		798 (721–1171)
Days of follow-up, range		36–1903
Status at last visit***	2nd transplant	1 (4.8)
	In remission - alive	16 (76.2)
	In remission - TRM	1 (4.8)
	Relapse - alive	2 (9.5)
	Relapse - DRM	1 (4.8)

ATG, antithymocyte globulin; BM, bone marrow; CR, complete remission; DLI, donor lymphocyte infusion; DRM, disease-related mortality; HLA, human leukocyte antigen; IQR,

interquartile range; MDS/MPN, myelodysplastic syndrome/myeloproliferative neoplasm; PBSC, peripheral blood stem cell; PR, partial remission; TBI, total body irradiation; TRM, transplantation-related mortality.

* Or accelerated phase

** Primary refractory or blast phase

*** Last visit between 2019 and 2022

Table B Characteristics of acute and late acute GvHD in children

		Children n=21 (%)
aGvHD	aGvHD	6 (28.6)
	No aGvHD	15 (71.4)
Grade of aGvHD at onset	1	5 (83.3)
	2	1 (16.7)
	3	0 (0)
	4	0 (0)
Grade of aGvHD at max	1	4 (66.7)
	2	1 (16.7)
	3	1 (16.7)
	4	0 (0)
Steroid-refractory aGvHD		4 (66.7)
laGvHD	laGvHD	1 (4.8)
	No laGvHD	20 (95.2)
laGvHD onset	Late onset de novo	0 (0)
	Persistent	1 (100)
	Recurrent	0 (0)
Grade of laGvHD severity at onset	1	0 (0)
	2	0 (0)
	3	1 (100)
	4	0 (0)
Grade of laGvHD at maximum severity	1	0 (0)
	2	0 (0)
	3	0 (0)
	4	1 (100)
Steroid-refractory laGvHD		1 (100)
Steroid-sensitive laGvHD		0 (0)

aGvHD, acute graft-versus-host disease; laGvHD, late aGvHD.

Table C cGvHD characteristics in children

Total		Children n=21 (%)
cGvHD	cGvHD	2 (9.5)
	No cGvHD	19 (90.5)
cGvHD type of onset	De novo	0 (0)
	Quiescent	1 (50)
	Progressive	1 (50)
cGvHD classification	Classic	1 (50)
	Overlap	1 (50)
Grade of cGvHD severity at onset	Mild	0 (0)
	Moderate	1 (50)
	Severe	1 (50)
Days from tx to cGvHD max symptoms, median (IQR)		247 (221–273)
Days from tx to cGvHD max symptoms, range		221–273
Platelets <100/nl at onset		1 (50)
Second-line therapy required		1 (50)
Systemic immunosuppression at onset of cGvHD	Yes	1 (50)
	No	1 (50)

cGvHD, chronic graft-versus-host disease; IQR, interquartile range; Tx, transplantation.

Supplementary Tables

Table 1 Use of GvHD prophylaxis in different donor constellations of total adult population and influence on development of cGvHD

Donor	GvHD prophylaxis	no cGvHD		cGvHD		Total	
		n	%	n	%	n	%
**Haplo**		**27**	**73.0**	**10**	**27.0**	**37**	**100**
	Cy	25	71.4	10	28.6	35	100
	Standard*	2	100.0		0.0	2	100
**Identical**		**35**	**55.6**	**28**	**44.4**	**63**	**100**
	ATG	13	86.7	2	13.3	15	100
	Cy	2	100.0		0.0	2	100
	Standard*	19	42.2	26	57.8	45	100
	Other	1	100.0		0.0	1	100
**Unrelated**		**114**	**58.2**	**82**	**41.8**	**196**	**100**
	ATG	79	69.3	35	30.7	114	100
	Cy	7	53.9	6	46.2	13	100
	Standard*	27	40.9	39	59.1	66	100
	Other	1	33.3	2	66.7	3	100
**Grand Total**		**176**	**59.5**	**120**	**40.5**	**296**	**100**

GvHD, graft-versus-host disease; cGvHD, chronic graft-versus-host disease; Cy, cyclophosphamide; ATG, antithymocyte globulin.

*standard prophylaxis: calcineurin inhibitors, cyclosporine (CSP) or tacrolimus, and methotrexate (MTX) or mycophenolate mofetil (MMF)

Table 2 laGvHD organ stages

	Organ specific severity					
**Adult**	**4**	**3**	**2**	**1**	**0**	**Total**
**STAGE 1**			2 (28.6%)	5 (71.4%)	7 (100%)	7 (100%)
Skin			2 (28.6%)	5 (71.4%)		7 (100%)
Liver					7 (100%)	7 (100%)
GI					7 (100%)	7 (100%)
**STAGE 2**		4 (33.3%)	3 (25.0%)	6 (50.0%)	12 (100%)	12 (100%)
GI			2 (16.7%)	6 (50.0%)	4 (33.3%)	12 (100%)
Skin		4 (33.3%)	1 (8.3%)	1 (8.3%)	6 (50.0%)	12 (100%)
Liver					12 (100%)	12 (100%)
**STAGE 3**		5 (55.6%)	3 (33.3%)	1 (11.1%)	9 (100%)	9 (100%)
GI		5 (55.6%)	3 (33.3%)	1 (11.1%)		9 (100%)
Skin			2 (22.2%)		7 (77.8%)	9 (100%)
Liver					9 (100%)	9 (100%)
**STAGE 4**	3 (100%)		1 (33.3%)		3 (100%)	3 (100%)
GI	2 (66.7%)				1 (33.3%)	3 (100%)
Liver			1 (33.3%)		2 (66.7%)	3 (100%)
Skin	1 (33.3%)				2 (66.7%)	3 (100%)
**Grand Total**	3 (9.7%)	9 (29.0%)	9 (29.0%)	12 (38.7%)	31 (100%)	31 (100%)

laGvHD, late acute graft-versus-host disease; GI, gastrointestinal.

Table 3 Survival status at day 100

		Total n=317 (%)	Adults n=296 (%)	Adults at risk n=249 (%)	Pediatrics n=21 (%)
Status at day 100	Death TRM	27 (8.5)	26 (8.8)	(0)	1 (4.8)
	Death relapse	5 (1.6)	5 (1.7)	(0)	(0)
	Second transplant	3 (0.9)	3 (1)	(0)	(0)
	Relapse but alive >100 days	14 (4.4)	13 (4.4)	(0)	1 (4.8)
	Alive in remission	268 (84.5)	249 (84.1)	249 (100)	19 (90.5)

TRM, transplantation-related mortality.

Table 4 Organ involvement in cGvHD

	Adults at risk n=119	Pediatrics n=2	Total n=121
Mouth	52.1%	50.0%	52.1%
Skin	40.3%	100.0%	41.3%
Eyes	26.1%	50.0%	26.4%
Liver	25.2%	0.0%	24.8%
GI	12.6%	100.0%	14.0%
Joints and fascia	5.9%	0.0%	5.8%
Lungs	5.0%	0.0%	5.0%
Genital tract	3.7%	0.0%	3.7%

cGvHD, chronic graft-versus-host disease; GI, gastrointestinal.

Table 5 Detailed organ stages in cGvHD at onset

	Organ specific severity											
	**3**		**2**		**1**		**0**		**NA**		**Total**	
**Mild cGvHD (n=60)**	n	(%)	n	(%)	n	(%)	n	(%)	n	(%)	n	(%)
Eyes	0	(0)	0	(0)	8	(13.3)	52	(86.7)	0	(0)	60	(100)
GI	0	(0)	0	(0)	5	(8.3)	55	(91.7)	0	(0)	60	(100)
Genital Tract	0	(0)	0	(0)	1	(1.7)	55	(91.7)	4	(6.7)	60	(100)
Joints and Fascia	0	(0)	0	(0)	2	(3.3)	58	(96.7)	0	(0)	60	(100)
Liver	0	(0)	0	(0)	9	(15)	51	(85.0)	0	(0)	60	(100)
Lungs	0	(0)	0	(0)		0 (0)	60	(100)	0	(0)	60	(100)
Mouth	0	(0)	0	(0)	30	(50)	30	(50)	0	(0)	60	(100)
Skin	0	(0)	1	(1.7)	17	(28.3)	42	(70)	0	(0)	60	(100)
**Moderate cGvHD (n=48)**												
Eyes	0	(0)	9	(18.8)	10	(20.8)	29	(60.4)	0	(0)	48	(100)
GI	0	(0)	4	(8.3)	4	(8.3)	40	(83.3)	0	(0)	48	(100)
Genital Tract	0	(0)	0	(0)	1	(2.1)	42	(87.5)	5	(10.4)	48	(100)
Joints and Fascia	0	(0)	3	(6.3)	1	(2.1)	44	(91.7)	0	(0)	48	(100)
Liver	0	(0)	9	(18.8)	8	(16.7)	31	(64.6)	0	(0)	48	(100)
Lungs	0	(0)	1	(2.1)	4	(8.3)	43	(89.6)	0	(0)	48	(100)
Mouth	0	(0)	10	(20.8)	17	(35.4)	21	(43.8)	0	(0)	48	(100)
Skin	0	(0)	16	(33.3)	7	(14.6)	25	(52.1)	0	(0)	48	(100)
**Severe cGvHD (n=11)**												
Eyes	0	(0)	3	(27.3)	1	(9.1)	7	(63.6)	0	(0)	11	(100)
GI	1	(9.1)	1	(9.1)		0 (0)	9	(81.8)	0	(0)	11	(100)
Genital Tract	1	(9.1)	1	(9.1)		0 (0)	7	(63.6)	2	(18.2)	11	(100)
Joints and Fascia	0	(0)	0	(0)	1	(9.1)	10	(90.9)	0	(0)	11	(100)
Liver	2	(18.2)	1	(9.1)	1	(9.1)	7	(63.6)	0	(0)	11	(100)
Lungs	1	(9.1)	0	(0)		0 (0)	10	(90.9)	0	(0)	11	(100)
Mouth	1	(9.1)	1	(9.1)	3	(27.3)	6	(54.5)	0	(0)	11	(100)
Skin	5	(45.5)	1	(9.1)	1	(9.1)	4	(36.4)	0	(0)	11	(100)
**Grand Total**	**10**	**(8.4)**	**48**	**(40.3)**	**91**	**(76.5)**	**119**	**(100)**	**11**	**(9.2)**	**119**	**(100)**

cGvHD, chronic graft-versus host disease; GI, gastrointestinal tract.

Table 6 Development of GvHD after DLI

DLI	GvHD	Adults at risk n=249 (%)	Adults total n=296 (%)
No DLI	Total	215 (86.3)	258 (87.2)
DLI	Total	34 (13.7)	38 (12.8)
	no aGvHD	24 (70.6)	28 (73.7)
	aGvHD	10 (29.4)	10 (26.3)
	no laGvHD	23 (67.6)	27 (71.1)
	laGvHD	11 (32.4)	11 (28.9)
	no cGvHD	24 (70.6)	28 (73.7)
	cGvHD	10 (29.4)	10 (26.3)

aGvHD, acute GvHD; cGvHD, chronic GvHD; DLI, donor lymphocyte infusion; GvHD; graft-versus-host disease.

Table 7 Single-agent therapy of cGvHD

cGvHD treatment		Adults n=33	%	Pediatrics n=1	%	Total n=34	%
Single-agent therapy		33	(100)	1	(100)	34	(100)
	Steroids	30	(90.9)		(0)	30	(88.2)
	MTX	2	(6.1)		(0)	2	(5.9)
	CSP	1	(3)		(0)	1	(2.9)
	Ruxolitinib		(0)	1	(100)	1	(2.9)

cGvHD, chronic graft-versus-host disease; CSP, cyclosporine; MTX, methotrexate.

Table 8 Combinations of double-agent therapy of cGvHD

cGvHD treatment		Adults at risk n=53	%	Total n=53	%
Double-agent therapy		53	(100)	53	(100)
	Steroids + CSP	30	(56.6)	30	(56.6)
	Steroids + Tacrolimus	11	(20.8)	11	(20.8)
	Steroids + MTX	3	(5.7)	3	(5.7)
	Steroids + Ruxolitinib	2	(3.8)	2	(3.8)
	Steroids + Ibrutinib	2	(3.8)	2	(3.8)
	Steroids + Everolimus	2	(3.8)	2	(3.8)
	Steroids + CSP + UVB	1	(1.9)	1	(1.9)
	CSP + Ruxolitinib	1	(1.9)	1	(1.9)
	CSP + MTX	1	(1.9)	1	(1.9)

cGvHD, chronic graft-versus-host disease; CSP, cyclosporine; MTX, methotrexate; UVB, ultraviolet B.

Table 9 Combinations of triple-agent therapy of cGvHD

cGvHD treatment		Adults n=7	%	Pediatrics n=1	%	Total n=8	%
Triple-agent therapy		7	(100)	1	(100)	8	(100)
	Steroids + Tacrolimus + ECP	2	(28.6)		(0)	2	(25)
	Steroids + Tacrolimus + Ibrutinib + Rituximab	1	(14.3)		(0)	1	(12.5)
	Steroid + ECP + Cyclophosphamide	1	(14.3)		(0)	1	(12.5)
	Steroids + Tacrolimus + ECP + Imatinib		(0)	1	(100)	1	(12.5)
	Steroids + Tacrolimus + MMF	1	(14.3)		(0)	1	(12.5)
	Steroids + CSP + Ruxolitinib + MTX	1	(14.3)		(0)	1	(12.5)
	Steroids + ECP + Ruxolitinib	1	(14.3)		(0)	1	(12.5)

cGvHD, chronic graft-versus-host disease; CSP, cyclosporine; ECP, extracorporeal photopheresis; MMF, mycophenolate mofetil; MTX, methotrexate.

Table 10 Outcome with second-line therapy

Status at last contact	No cGvHD n=130 (%)	cGvHD First-line therapy n=85 (%)	Second-line therapy n=34 (%)
Alive	86 (66.2)	65 (76.5)	29 (85.3)
Second transplant	16 (12.3)	4 (4.7)	0 (0)
Alive in remission	54 (41.5)	48 (56.5)	27 (79.4)
Alive with relapse	4 (3.1)	3 (3.5)	0 (0)
Lost to follow-up	12 (9.2)	10 (11.8)	2 (5.9)
Dead	44 (33.8)	20 (23.5)	5 (14.7)
Died due to relapse	19 (14.6)	10 (11.8)	1 (2.9)
TRM	25 (19.2)	10 (11.8)	4 (11.8)

cGvHD, chronic graft-versus-host disease; TRM, transplantation-related mortality

Supplementary Figures

Fig. 1 aGvHD organ stages

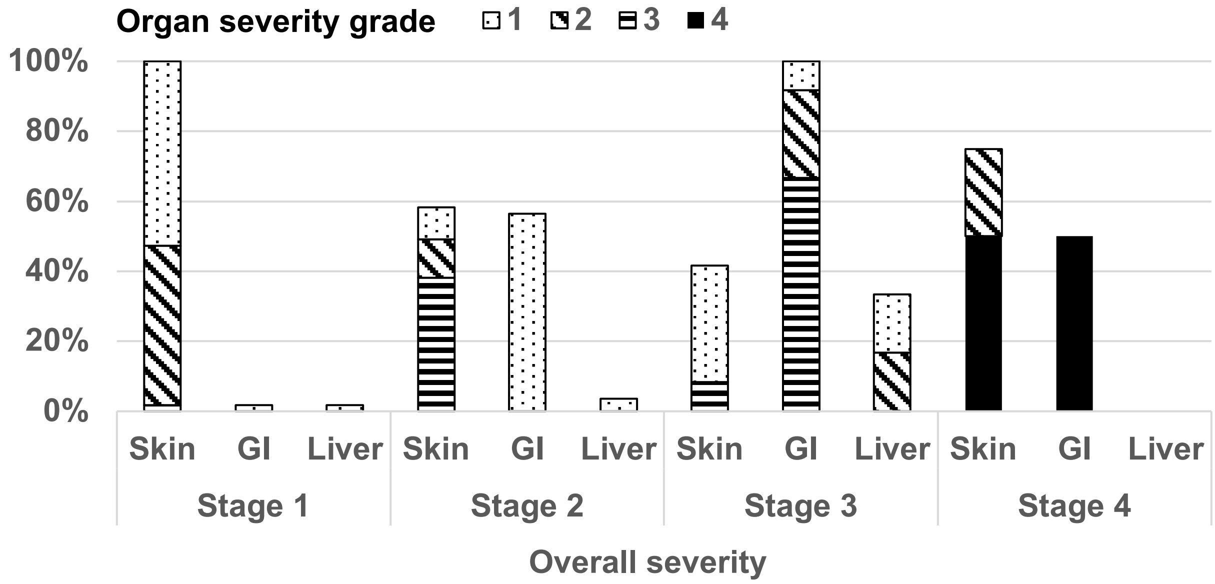

aGvHD, acute graft-versus-host disease; GI, gastrointestinal.

Fig. 2 laGvHD organ stages

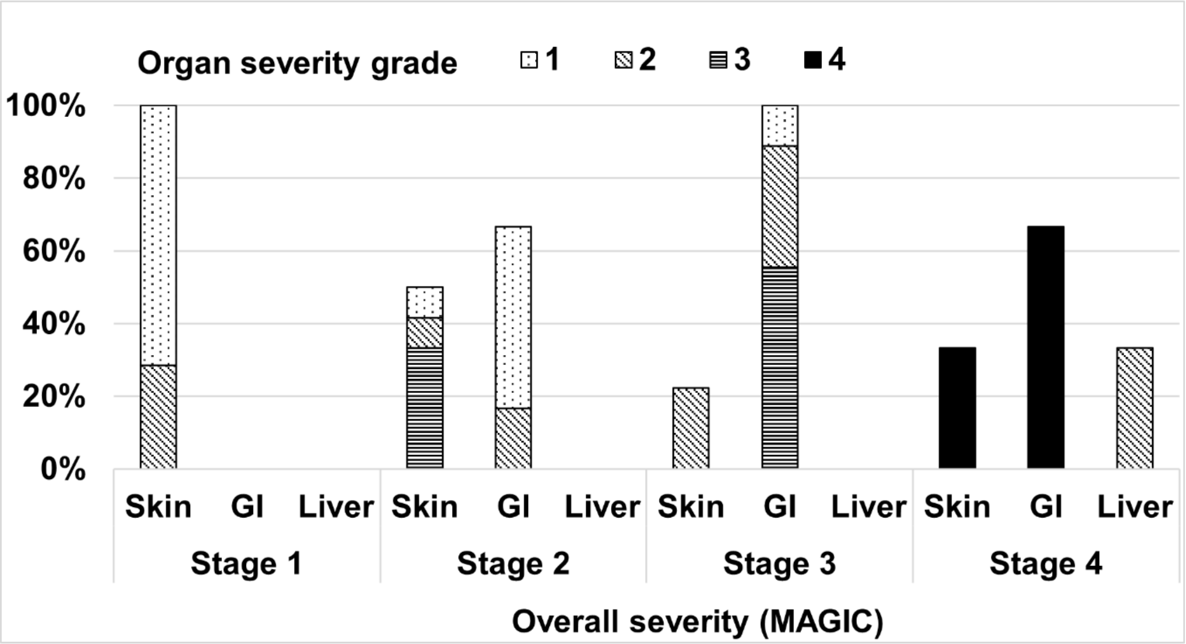

GI, gastrointestinal; laGvHD, late acute graft-versus-host disease.

Fig. 3 Organ severity at onset of cGvHD in adults at risk

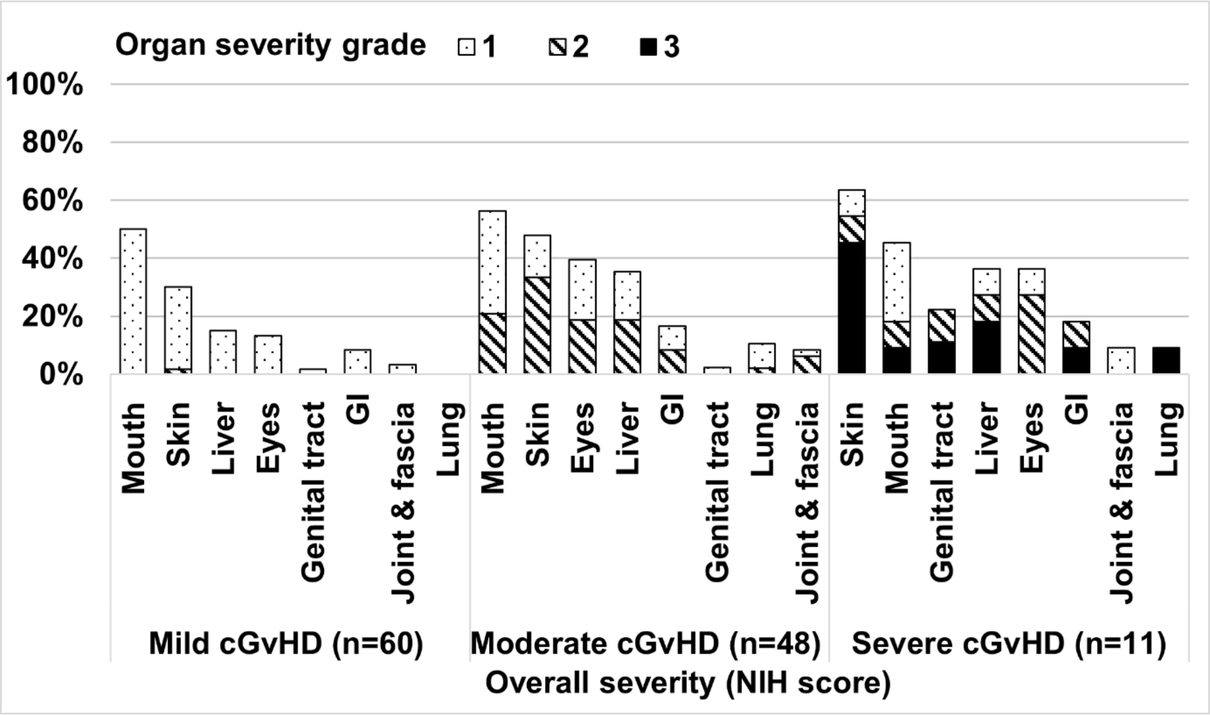

cGvHD, chronic graft-versus-host disease; GI, gastrointestinal; NIH, National Institutes of Health.

Fig. 4 CI of cGvHD in patients at risk (n=249)

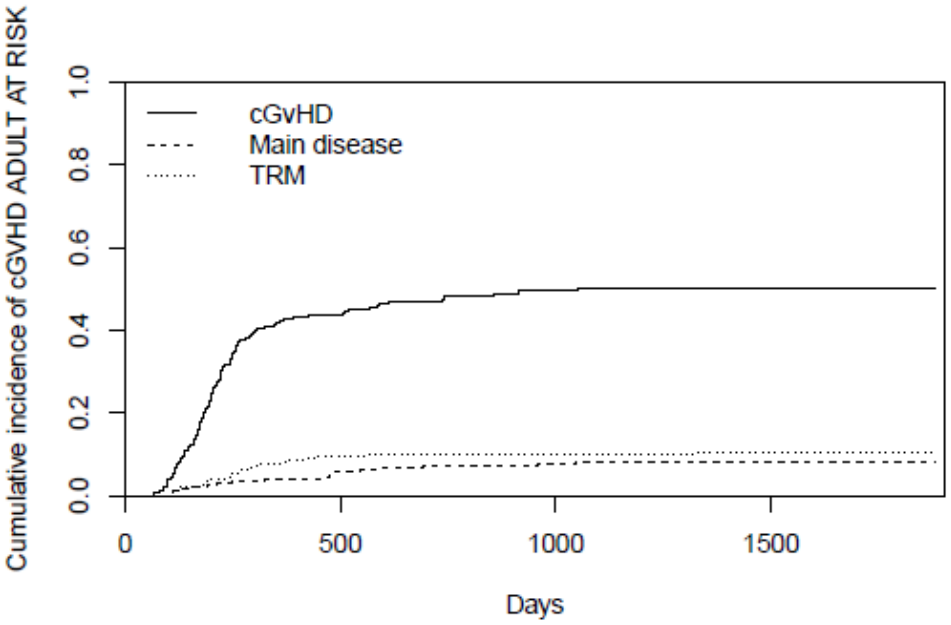

cGvHD, chronic graft-versus-host disease; CI, cumulative incidence; TRM, transplantation-related mortality.

Fig. 5 TRM, R and OS in patients with and without laGvHD

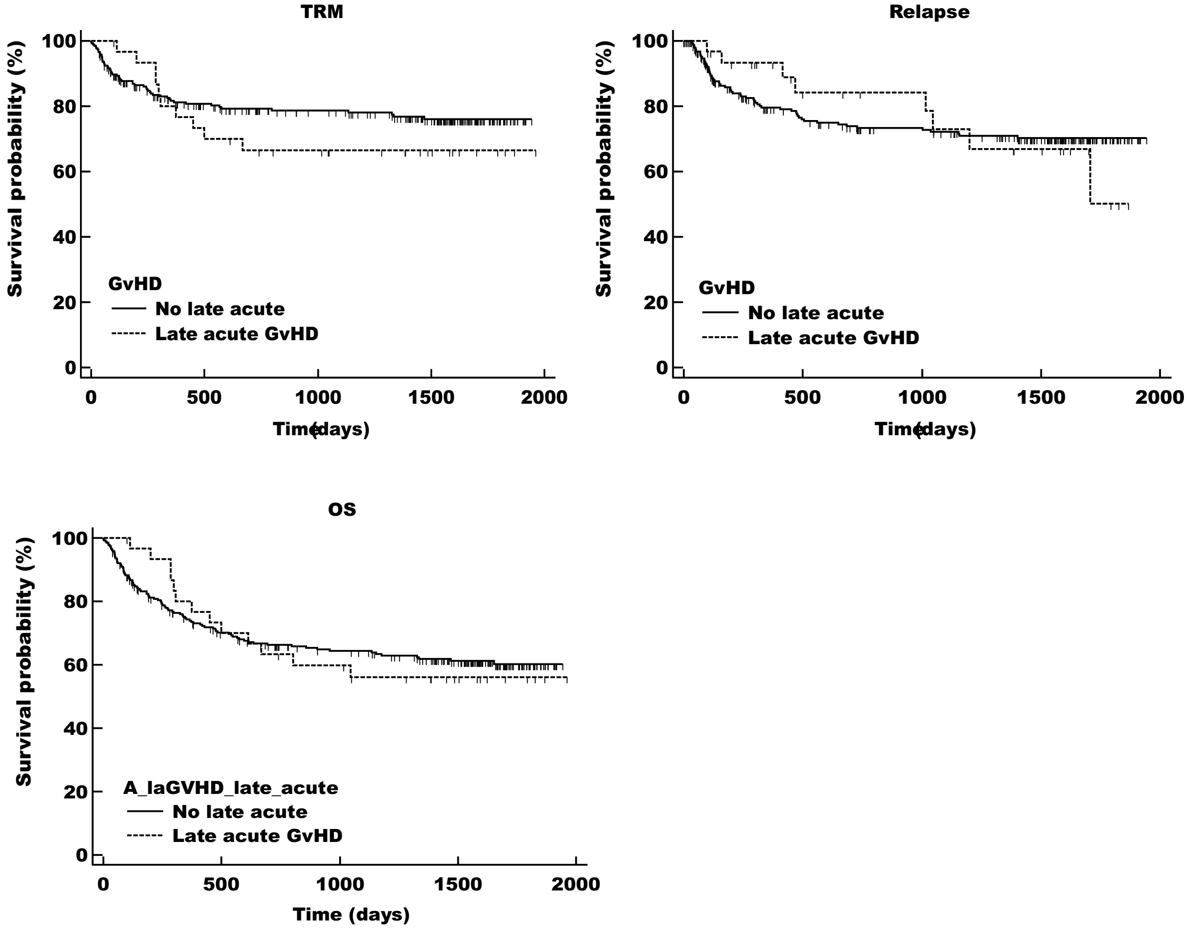

TRM, transplantation-related mortality; R, relapse; OS, overall survival; laGvHD, late acute graft-versus-host disease.

Fig. 6 Global score at start of second-line therapy

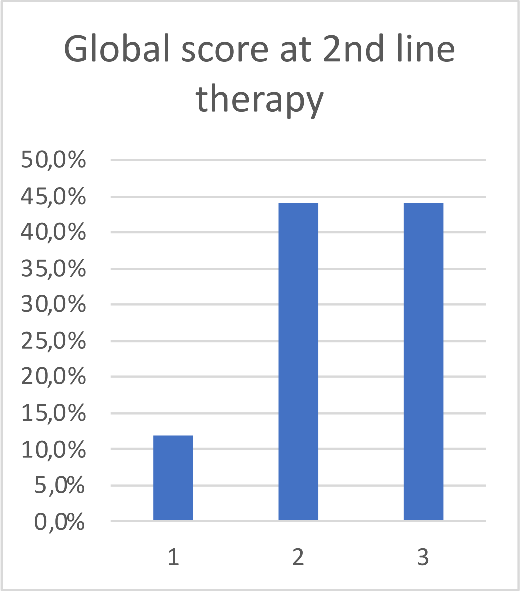

1=mild cGvHD; 2=moderate cGvHD; 3=severe cGvHD;

cGvHD, chronic graft-versus-host disease.

Fig. 7 Need of second-line therapy in different severities of cGvHD at onset

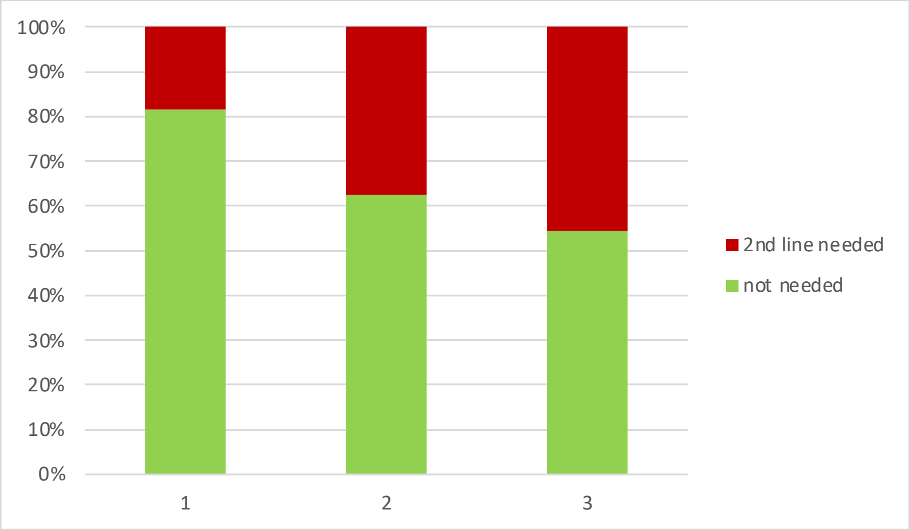

1=mild cGvHD; 2=moderate cGvHD; 3=severe cGvHD;

cGvHD, chronic graft-versus-host disease.

Fig. 8a TRM in patients with and without DLI

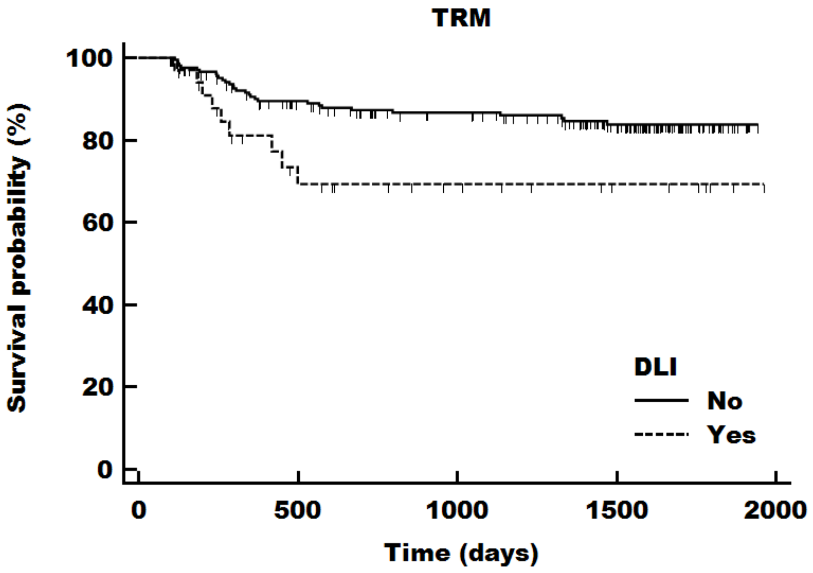

DLI, donor lymphocyte infusion; TRM, transplantation-related mortality.

We found significantly lower TRM rates in patients who received donor lymphocyte infusion (DLI) compared to patients without DLI (26.5% vs 14.0%; p=0.0179).

Fig. 8b TRM in the population at risk according to onset severity grade of cGvHD

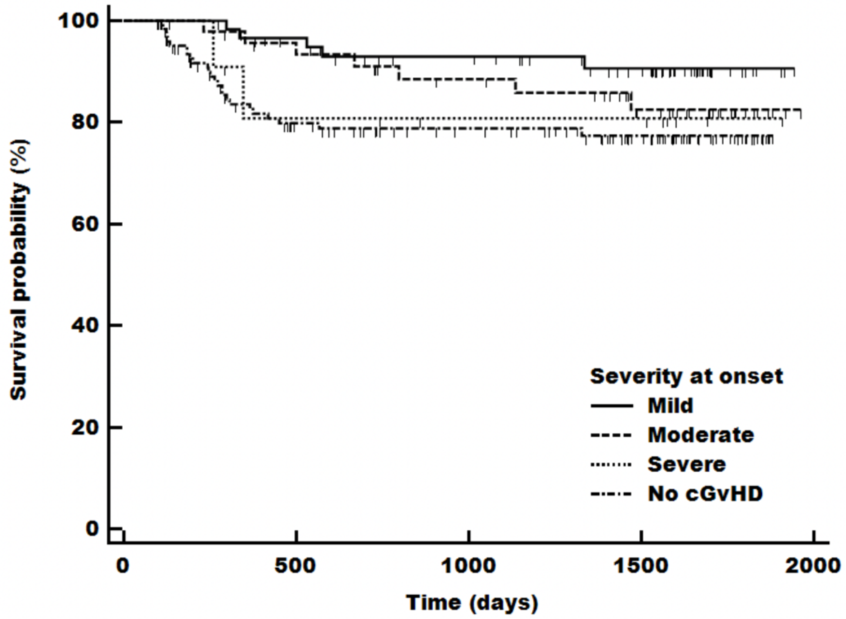

TRM, transplantation-related mortality; cGvHD, chronic graft-versus-host disease.

Fig. 9 Cumulative incidence of relapse with TRM as competing risk according to the overall cGvHD diagnosis (a), cGvHD severity (b). To make the plots easier to visualize, competing risk (TRM) is only shown on panel A. Furthermore, panel B is limited to cGvHD patients only, as cGvHD significantly affected relapse.

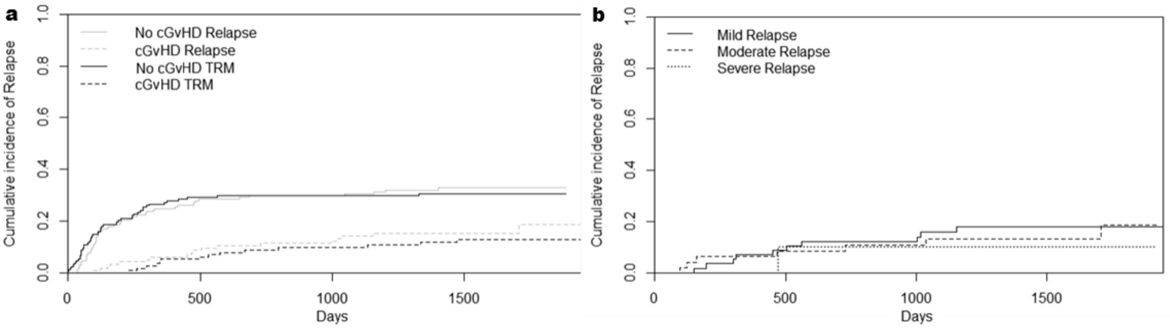

cGvHD, chronic graft-versus-host disease; TRM, transplantation-related mortality.

Fig. 10 OS of patients depending on type of GvHD (a) in the total adult population, severity of cGvHD at onset (b) in the population at risk, cGvHD classification (c) and transplant cell source (d) in the total adult population

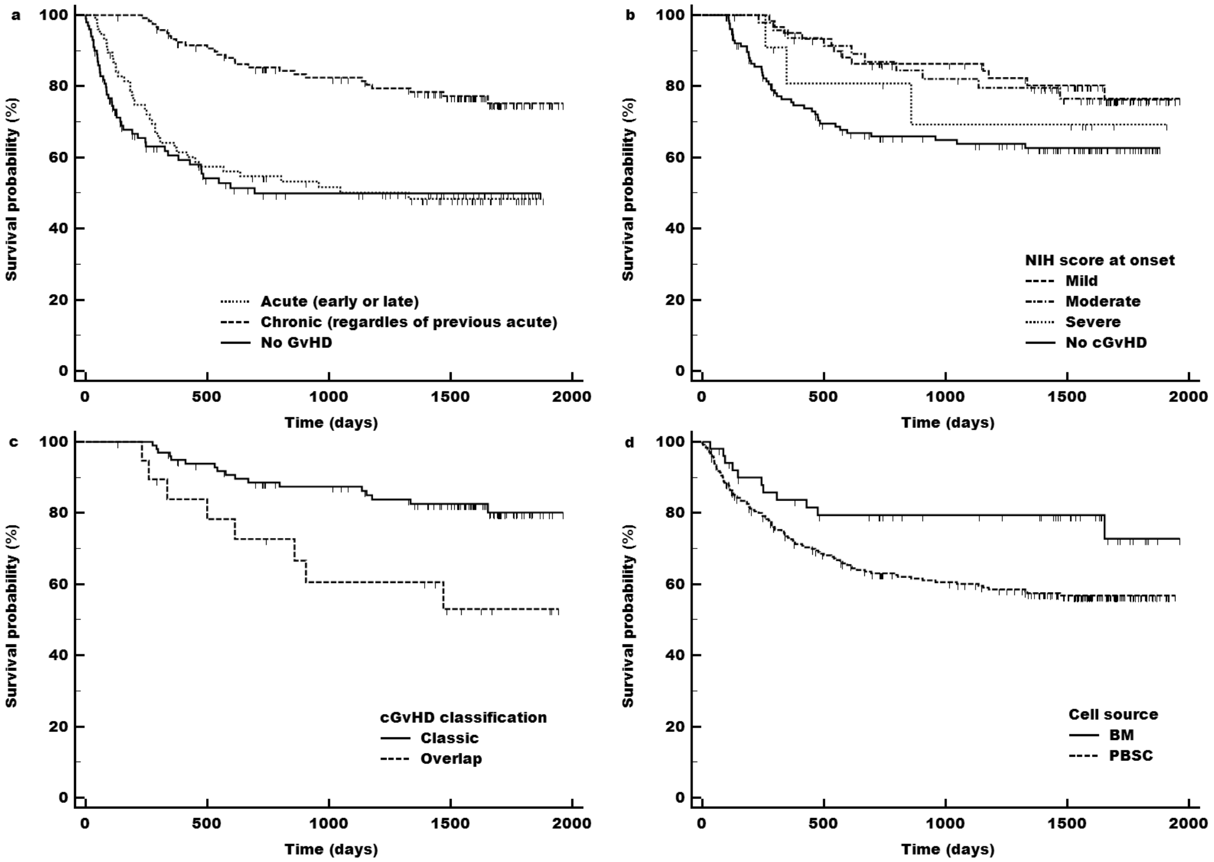

BM, bone marrow; cGvHD, chronic host-versus host disease; NIH, National Institutes of Health, PBSC, peripheral blood stem cell

Fig. 11 Overall survival according to patient age in the total population (A; n=317) or without pediatric patients (B; N=296), steroid-refractory aGvHD (C), or relapse with or without cGvHD diagnosis (D).

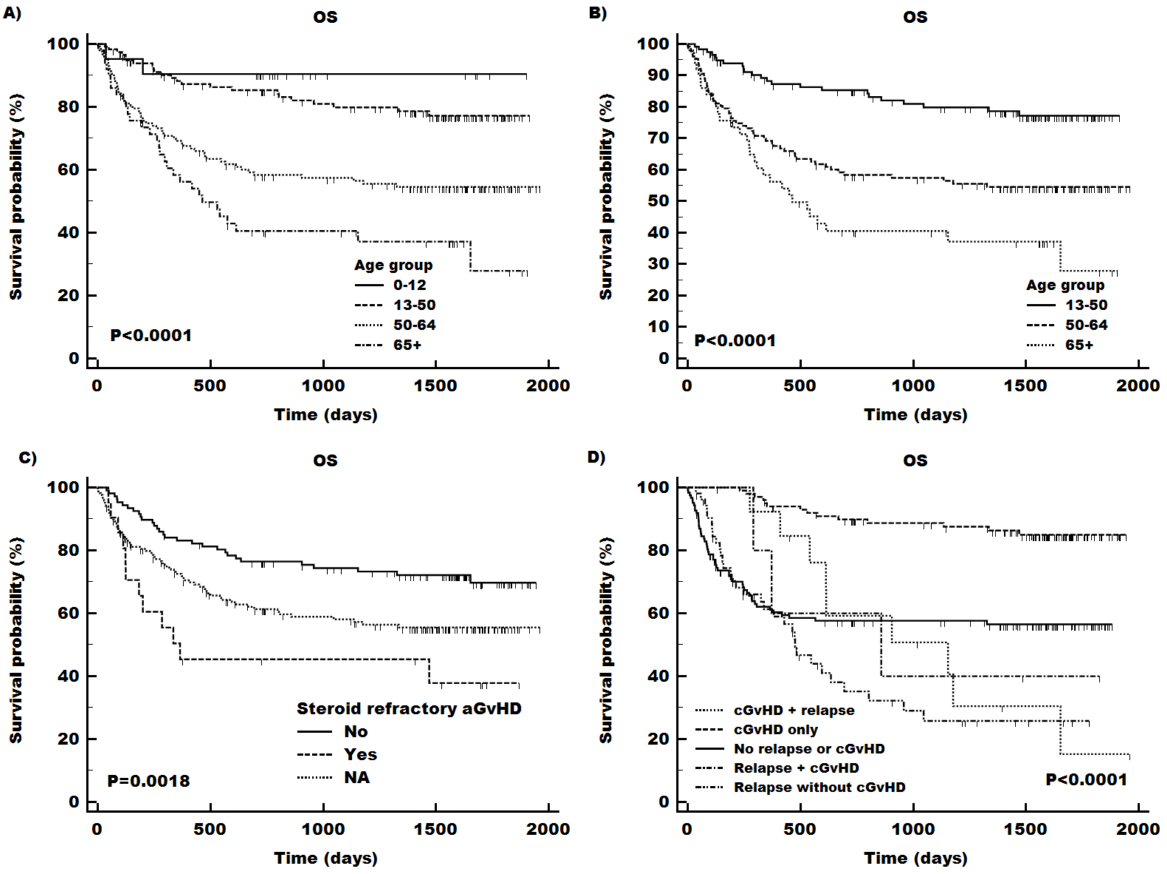

aGvHD, acute graft-versus-host disease; cGvHD, chronic graft-versus-host disease; OS, Overall survival.

As expected, the age of patients correlated significantly with overall survival (OS); the younger the patients, the better the outcome, with or without taking pediatric patients into consideration (both $p<0.0001$). Expectations were also met for patients with steroid-refractory acute graft-versus-host disease (aGvHD) ($p=0.0018$) as well as relapse independent of previous or subsequent chronic graft-versus-host disease (cGvHD) development ($p<0.001$). OS was low, regardless of whether cGvHD occurred prior to relapse, after relapse, or not at all (30.8% vs 40% vs 39.6%). This observation, however, was based on a limited number of patients.

Fig. 12 Landmark analysis of the total adult population applying the landmark on day 100 concerning TRM (a), DFS (b) and OS (c)

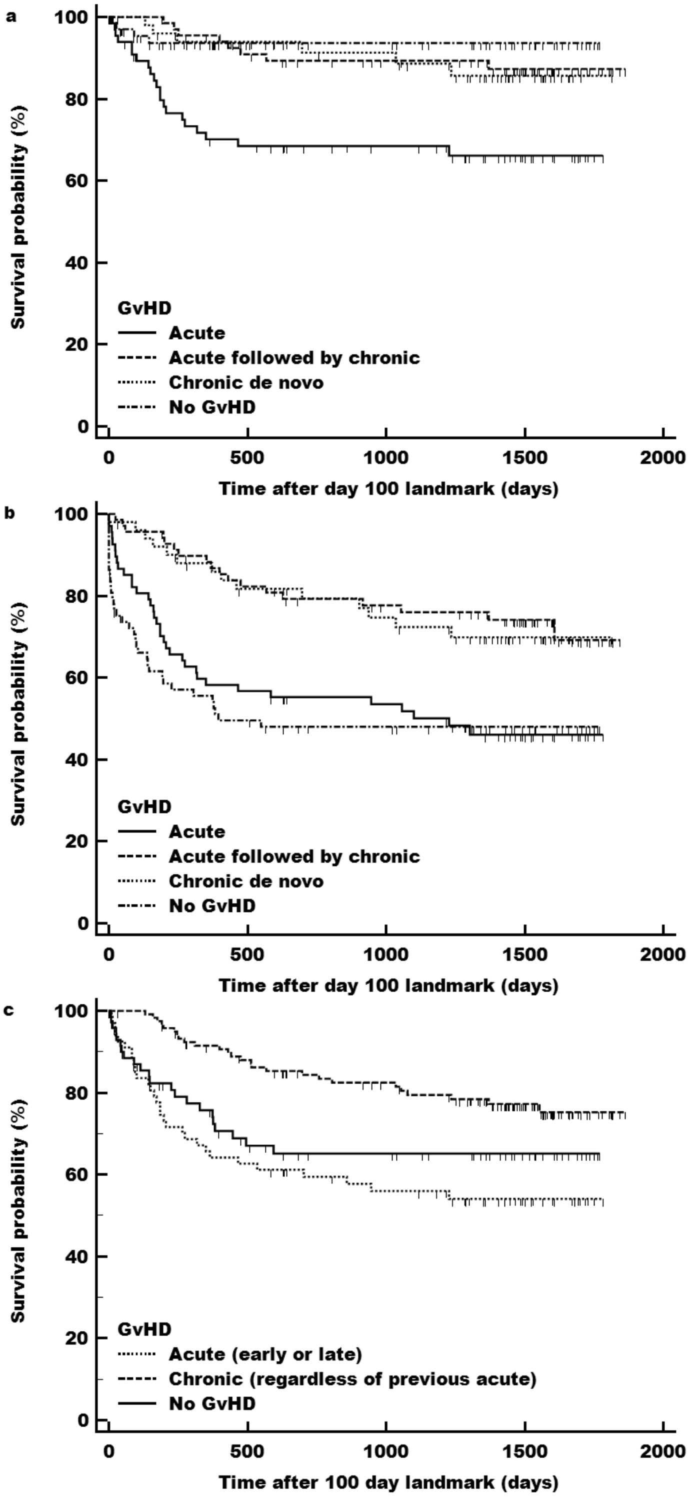

TRM, transplantation-related mortality; DFS, disease-free survival; OS, overall survival; GvHD, graft-versus-host disease.
